# Decompensated liver cirrhosis and neural regulation of mesenteric vascular tone in rats: role of sympathetic, nitrergic and sensory innervations

**DOI:** 10.1038/srep31076

**Published:** 2016-08-03

**Authors:** Esther Sastre, Laura Caracuel, Isabel Prieto, Pablo Llévenes, M. Ángeles Aller, Jaime Arias, Gloria Balfagón, Javier Blanco-Rivero

**Affiliations:** 1 Departamento de Fisiología, Facultad de Medicina, Universidad Autónoma de Madrid, España; 2 Instituto de Investigación Sanitaria del Hospital Universitario La Paz (IdiPAZ), Madrid, España; 3 Departamento de Cirugía General y Digestiva, Hospital la Paz, Madrid, España; 4 Cátedra de Cirugía, Facultad de Medicina, Universidad Complutense de Madrid, España.

## Abstract

We evaluated the possible alterations produced by liver cholestasis (LC), a model of decompensated liver cirrhosis in sympathetic, sensory and nitrergic nerve function in rat superior mesenteric arteries (SMA). The vasoconstrictor response to electrical field stimulation (EFS) was greater in LC animals. Alpha-adrenoceptor antagonist phentolamine and P2 purinoceptor antagonist suramin decreased this response in LC animals more than in control animals. Both non-specific nitric oxide synthase (NOS) L-NAME and calcitonin gene related peptide (CGRP) (8-37) increased the vasoconstrictor response to EFS more strongly in LC than in control segments. Vasomotor responses to noradrenaline (NA) or CGRP were greater in LC segments, while NO analogue DEA-NO induced a similar vasodilation in both experimental groups. The release of NA was not modified, while those of ATP, nitrite and CGRP were increased in segments from LC. Alpha 1 adrenoceptor, Rho kinase (ROCK) 1 and 2 and total myosin phosphatase (MYPT) expressions were not modified, while alpha 2B adrenoceptor, nNOS expression and nNOS and MYPT phosphorylation were increased by LC. Together, these alterations might counteract the increased splanchnic vasodilation observed in the last phases of decompensated liver cirrhosis.

Liver cirrhosis is a common disease currently ranked as one of the 10 most common causes of death in the Western world (Stewart and Day, 2003). It courses with portal hypertension (PH) related to augmented hepatic vascular resistance, caused by an increase in vasoconstrictor factors like thromboxane A_2_ (TXA_2_)^1^, and a reduction in nitric oxide (NO) bioavailability^2^. This pathology is also associated with the development of hyperdynamic circulation^3^, in which peripheral arterial vasodilation, mainly in the splanchnic circulation, plays a major role^4^. This decreased splanchnic vascular resistance is associated to alterations in different endothelial factors in these vessels, such as increased release and sensitivity of vasodilator factors like NO^5^,^6^ and prostaglandin I_2_ (PGI_2_)^7^,^8^ as well as decreased response to factors like noradrenaline (NA)^9^, phenylephrine or U-46619^10^,^11^. Endothelial factor participation is known to change over time in the course of liver cirrhosis. Thus, although the increase in splanchnic and systemic circulation is maintained in decompensated liver cirrhosis and long-term PH models, NO involvement decreases, leading to greater participation by PGI_2_^12^,^13^,^14^,^15^.

Apart from endothelial factors, vascular tone is also determined by the participation of perivascular innervation. Rat mesenteric arteries possess rich sympathetic^16^, nitrergic^17^,^18^ and sensory^19^ innervations, which respectively release NA and ATP, NO and calcitonin gene related peptide (CGRP), when electrically stimulated. These neurotransmitters participate in vasomotor tone modulation through their vasoconstrictor and vasodilator actions on smooth muscle cells. We have reported that different physiopathological situations, such as aging^20^,^21^, hypertension^22^,^23^ or diabetes^24^,^25^ can modify the participation of the different neurotransmitters involved in the maintenance of vascular tone. In line with this, in a model of mild liver cirrhosis induced by carbon tetrachloride treatment, several studies have reported decreased sympathetic innervation function^26^,^27^,^28^,^29^,^30^, increased NO release from neuronal NO synthase (nNOS)^29^,^31^ and augmented sensory innervation function^32^. Additionally, we have previously described a rearrangement of participation by the different components of perivascular mesenteric innervation in a model of long-term PH^21^. However, to the best of our knowledge, possible alterations in the function of perivascular innervation components in decompensated liver cirrhosis have yet to be studied.

In light of this background, the aim of this study was to analyse the possible alterations in the vasomotor response to electric field stimulation (EFS) in rat superior mesenteric artery, and how sympathetic, sensory and nitrergic components could be affected in decompensated liver cirrhosis induced by liver cholestasis.

## Materials and Methods

### Animals

Male Wistar rats were obtained and housed in the Animal Facility of the Universidad Autónoma de Madrid (Registration number EX-021U). The research conforms to the European Commission Directive 86/609 CEE Art. 21 (1995), and the Guide for the Care and Use of Laboratory Animals published by the US National Institutes of Health (NIH Publication No. 85-23, revised 1996). This study has been approved by the ethical committee of the Universidad Autónoma de Madrid.

Rats (Initial weight: 294.5 ± 2.6 g) were divided into two groups: Sham-operated (SO; n = 10), in which the common bile duct was dissected; and microsurgical liver cholestasis (LC; n = 10), in which the extrahepatic biliary tract was resected^33^,^34^. Surgery was performed under aseptic but not sterile conditions. Briefly, rats were anaesthetised with Ketamine hydrochloride (100 mg/kg) and Xylazine (12 mg/kg) i.m. In the SO group, the bile duct and its lobular branches were dissected. In the LC group, the extrahepatic bile tract was resected using a binocular operatory microscope (Zeiss, OPMI 1-FR). First, the common bile duct was ligated (silk 4/0) and sectioned close to the beginning of its intrapancreatic portion. Dissection and sectioning between ligatures of all biliary branches that drain the hepatic lobes is possible using a binocular operatory microscope (Zeiss, OPMI 1-FR). The dissection and section of the bile ducts from the four liver lobes of the rat must be done without injuring either the portal or, and most especially, the arterial vascularization of these lobes. The abdomen was closed in two layers by continuous running sutures using an absorbable suture (3/0 polyglycolic acid) and silk (3/0). Buprenorphine s.c. (0.05 mg/kg/8 hours) was administered postoperatively for analgesia the first 24 hours after the surgery.

Rats were housed at a constant room temperature, humidity and 12 h light/dark cycle and had free access to tap water and standard rat chow. Mean arterial pressure (MAP) was measured using the tail-cuff method^21^,^32^, 8–9 weeks after the surgery was performed. Afterwards, animals were sacrificed by CO_2_ inhalation; ascetic liquid was collected, and liver, spleen and the first branch of the mesenteric artery were removed and placed in cold Krebs−Henseleit solution (KHS, in mmol/L: NaCl 115; CaCl_2_ 2.5; KCl 4.6; KH_2_PO_4_ 1.2; MgSO_4_∙7H_2_O 1.2; NaHCO_3_ 25; glucose 11.1, Na_2_ EDTA 0.03) at 4 °C.

### Portal Vein Pressure Measurement

Splenic pulp pressure, an indirect measurement of portal pressure (PP) was measured by inserting a fluid filled 20-gauge needle into the splenic parenchyma^15^,^21^,^32^. The needle was joined to a PE-50 tube, and then connected to a pressure recorder (PowerLab 200 ML 201) and to a transducer (Sensonor SN-844) with a Chart V 4.0 computer program (ADI Instruments); these were calibrated before each experiment. The pressure reading was considered satisfactory when a stable recording was produced and respiratory variations were observed. Previous studies have demonstrated the excellent correlation between splenic pulp pressure and PP^35^.

### Vascular Reactivity

The method used for isometric tension recording has been described in full elsewhere^18^,^36^. Mesenteric segments from SO and LC animals were suspended in an organ bath containing 5 mL of KHS at 37 °C and continuously bubbled with a 95% O_2_ to 5% CO_2_ mixture (pH of 7.4); two parallel stainless steel pins were introduced through the lumen of the vascular segment: one was fixed to the bath wall, and the other connected to a force transducer (Grass FTO3C; Quincy, Mass., USA); this, in turn, was connected to a model 7D Grass polygraph. Some experiments were performed in endothelium-denuded segments to eliminate the main source of vasoactive substances, including endothelial NO. This avoided possible actions by different drugs on endothelial cells that could lead to result misinterpretation. Endothelium was removed by gently rubbing the luminal surface of the segments with a thin wooden stick. The segments were subjected to a tension of 0.5 g, which was readjusted every 15 min during a 90-min equilibration period before drug administration. After this, the vessels were exposed to 75 mmol/L KCl to check their functional integrity. Endothelium removal did not alter the contractions elicited by 75 mmol/L KCl. After a washout period, the presence or absence of vascular endothelium was tested by the inability of 10 μmol/L acetylcholine (Ach) to relax segments precontracted with NA. Arteries which relaxed either more than 90% or less than 10% of the previous tone obtained by NA were respectively considered to be endothelium-intact or endothelium-denuded.

For electrical field stimulation (EFS) experiments, segments were mounted between two platinum electrodes 0.5 cm apart and connected to a stimulator (Grass, model S44) modified to supply adequate current strength. The parameters used for EFS were 200 mA, 0.3 ms, with ascending frequencies from 1 to 16 Hz, for 30 s at each frequency, at an interval of 1 min between each stimulus, the time required to recover basal tone. To prove the neuronal origin of the EFS-induced contractile response, segments were incubated with nerve impulse blocker tetrodotoxin (TTX, 0.1 μmol/L). A washout period of at least 1 h was necessary to avoid desensitisation between consecutive curves. Two successive frequency-response curves separated by 1-hour intervals produced similar contractile responses.

To determine the participation of sympathetic innervation in the EFS-induced response in segments from the SO and LC animals, 1 μmol/L phentolamine, an alpha-adrenoceptor antagonist, 0.1 mmol/L suramin, a non-specific P2 purinergic receptor antagonist, or a combination of phentolamine plus suramin, were added to the bath 30 min before performing the frequency-response curve. Additionally, the vasoconstrictor response to exogenous NA (1 nmol/L-10 μmol/L) was tested in segments from both rat groups.

To analyse the participation of NO in the EFS-induced response in segments from SO and LC animals, 0.1 mmol/L Nω-nitro-L-arginine methyl ester (L-NAME), a non-specific inhibitor of nitric oxide synthase (NOS), was added to the bath 30 min before performing the second frequency–response curve. The vasodilator response to the NO donor, diethylamine NONOate, (DEA-NO, 0.1 nmol/L–0.1 mmol/L) was determined in NA-precontracted segments from the two groups.

To study the possible participation of sensory innervation in the EFS-induced response, 0.5 μmol/L CGRP (8-37), a CGRP receptor antagonist, was added to the bath 30 min before performing the second frequency–response curve. The vasodilator response to exogenous CGRP (0.1 nmol/L–0.1 μmol/L) was determined in NA-precontracted segments from both experimental groups.

### NA, ATP, nitrite and CGRP release

To measure the release of NA, ATP, nitrite and CGRP, we used a Noradrenaline research EIA (Labor Diagnostica Nord, Gmbh & Co., KG), an ATP Colorimetric/Fluorometric Assay kit (Abcam, Cambridge, UK), Nitric Oxide Assay Kit (Abcam, Cambridge, UK) and a rat CGRP enzyme immunoassay kit (Spibio, Bertin group), following the manufacturers’ instructions. For sample collecting, endothelium-denuded segments of rat mesenteric arteries from all experimental groups were preincubated for 30 minutes in 5 mL of KHS at 37 °C and continuously gassed with a 95% O_2_–5% CO_2_ mixture (stabilisation period). This was followed by two washout periods of 10 min in a bath of 0.4 mL of KHS. Then, the medium was collected to measure basal release. Next the organ bath was refilled and cumulative EFS periods of 30 s at 1, 2, 4, 8 and 16 Hz were applied at 1 min intervals. Afterwards, the medium was collected to measure EFS-induced neurotransmitter release. Samples were immediately frozen in liquid nitrogen and conserved at −70 °C until experiments were performed. The EFS-induced neurotransmitter release was calculated by subtracting basal release from that evoked by EFS. Results were expressed as pg NA/mL mg tissue, pmol ATP/mL mg tissue, pmol nitrite/mg tissue and pg CGRP/mL mg tissue.

### Western blot analysis

Western blot analysis was performed as previously described^37^. For these experiments, we used a mouse monoclonal antibody against nNOS (1:2000, BD Biosciences), a rabbit polyclonal antibody against nNOS phosphorylated In Ser1417 (P-nNOS, 1:2000, Abcam), a rabbit monoclonal antibody against Rho kinase 1 (ROCK1, 1:1000, Abcam), a mouse monoclonal antibody against ROCK2 (1:500, Abcam), a rabbit polyclonal antibody against myosin phosphatase (MYPT, 1:2000, Abcam), a rabbit polyclonal antibody against MYPT phosphorylated In Thr696 (P-MYPT, 1:500, Abcam), and a monoclonal anti-β-actin-peroxidase antibody (1:50000, Sigma-Aldrich, Spain). Appropriate positive controls were used for each blot.

### Drugs used

L -NA hydrochloride, ACh chloride, diethylamine NONOate diethyilammonium salt, 8-37 CGRP, rat CGRP, TTX, L -NAME hydrochloride, phentolamine, and suramin were from Sigma-Aldrich, Spain. Stock solutions (10 mmol/L) of drugs were made in distilled water, except for NA, which was dissolved in a NaCl (0.9%)-ascorbic acid (0.01% w/v) solution. These solutions were kept at –20 °C and appropriate dilutions were made in KHS on the day of the experiment.

### Data Analysis

The responses elicited by EFS and NA were expressed as a percentage of the initial contraction elicited by 75 mmol/L KCl for comparison between experimental groups. To determine differences in the effect of endothelium removal, phentolamine, suramin, L-NAME or CGRP(8–37) on the responses to EFS, we analysed the differences between areas under the curve (dAUC), which were expressed as the percentage of increase or decrease in the area under the curve produced by each drug. The relaxations induced by either DEA-NO or CGRP were expressed as a percentage of the initial contraction elicited by NA. Results are given as mean ± S.E.M. Statistical analysis was performed by comparing the curve obtained in the presence of the different substances with the previous or control curve by means of two-way analysis of variance (ANOVA) followed by a Fisher’s *post hoc* test, using GraphPad Prism 6.0 software (CA, USA). For dAUC and NA, ATP, nitrite and CGRP release data, the statistical analysis was done using Student’s t-test. P < 0.05 was considered significant.

## Results

### Animal evolution

All LC animals showed jaundice and choluria. Paraesophageal, splenorenal and pararectal collateral vessels developed in LC animals (Data not shown). Body weight gain was lower in LC animals. Diminished mean blood pressure, PH, spleen and liver hypertrophy, and extravasation of ascetic fluid were also present in LC animals (Table 1).

### Vasomotor Response to KCl

In endothelium-intact mesenteric segments, the vasoconstrictor response to 75 mmol/L KCl was similar in both experimental groups (SO: 1516 +53.9 mg; LC: 1541 + 82.8 mg; P > 0.05). Endothelium removal did not alter KCl-induced vasoconstriction (SO: 1505 + 83.8 mg; LC: 1477 + 65.8 mg; P > 0.05).

### Vascular Responses to EFS

**Figure 1:**
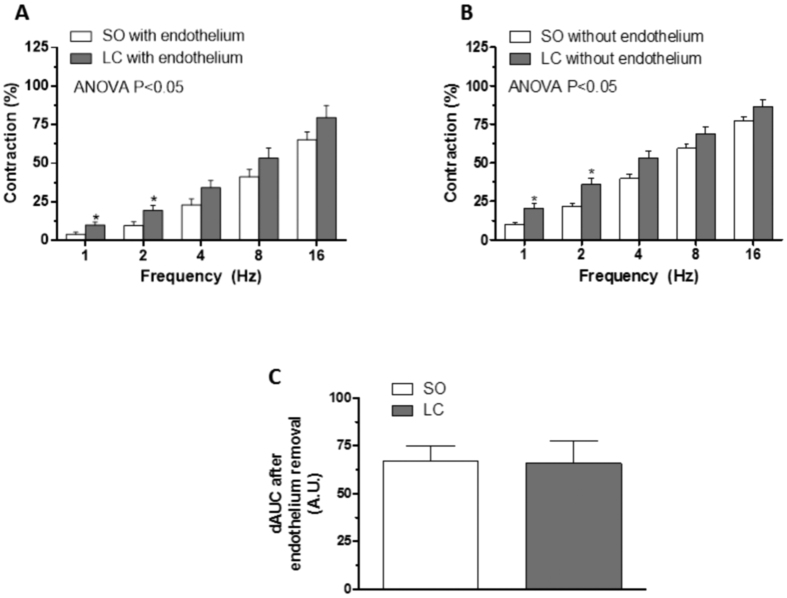
Vasoconstrictor response to EFS. EFS-induced vasoconstriction in mesenteric segments with (**A**) or without (**B**) endothelium from Sham-operated (SO) or liver cholestasis (LC) rats. Results (mean ± SEM) are expressed as a percentage of the initial contraction elicited by KCl. n = 10 animals per group. *P < 0.05 (Fisher *post hoc* test) (**C**) Differences of area under the curve (dAUC) in the absence or presence of endothelium. *P < 0.05 SO vs. LC rats (Student’s t-test). dAUC values are expressed as arbitrary units.

The application of EFS induced a frequency-dependent contractile response in endothelium-intact mesenteric segments from both the SO and LC groups. This vasoconstriction was greater in segments from LC rats compared to SO animals (Fig. 1A). Endothelium removal increased EFS-induced contractile response similarly in segments from both experimental groups (Fig. 1B,C). EFS-induced contractions were practically abolished in segments from both experimental groups by the neurotoxin TTX (0.1 mmol/L) (Table 2).

### Participation of the sympathetic component of mesenteric vascular innervation

**Figure 2:**
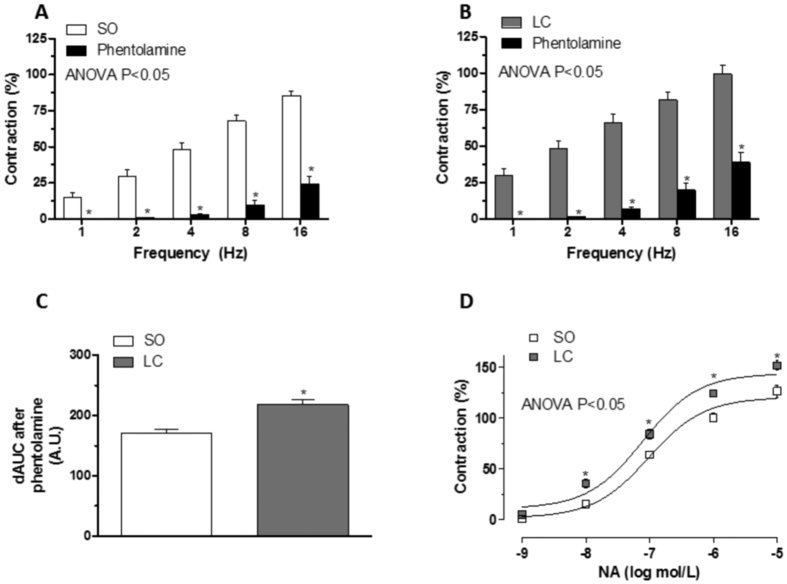
Effect of liver cholestasis on sympathetic innervation function. Effect of preincubation with 1 μmol/L phentolamine on vasoconstrictor response induced by EFS in endothelium-denuded mesenteric segments from Sham-operated (SO, **A**) and liver cholestasis (LC, **B**) rats. Results (mean ± S.E.M.) were expressed as a percentage of the initial contraction elicited by KCl. n = 6-8 animals per group. *P < 0.05 (Fisher *post hoc* test) (**C**) Differences of area under the curve (dAUC) in the absence or presence of 1 μmol/L phentolamine. *P < 0.05 SO vs. LC rats (Student’s t test). dAUC values are expressed as arbitrary units. (**D**) Vasoconstrictor response to NA in segments of SO and LC rats. Results (mean ± S.E.M.) were expressed as a percentage of the initial contraction elicited by KCl. n = 6 animals per group. *P < 0.05 (Fisher *post hoc* test).

Preincubation with the alpha-adrenergic antagonist phentolamine (1 μmol/L) decreased the vasoconstrictor response induced by EFS in endothelium-denuded segments from both rat groups (Fig. 2A,B). This decrease was greater in mesenteric segments from LC animals (Fig. 2C). NA-induced vasoconstriction was greater in mesenteric segments from the LC group than in segments from SO animals (Fig. 2D). EFS-induced NA release was similar in both groups (Table 3).

**Figure 3:**
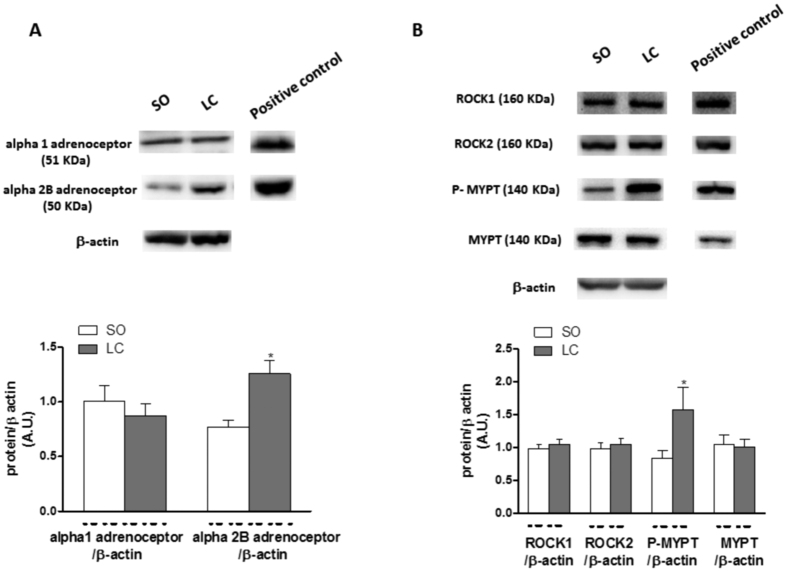
Effect of liver cholestasis on alpha adrenoceptor signalling transduction pathway. (**A**) Effect of liver cholestasis on alpha 1- adrenoceptor and alpha 2B- adrenoceptor expression. The blot is representative of six separate segments from each group. Rat brain homogenates were used as a positive control. Lower panel shows relation between alpha 1-adrenoceptor or alpha 2B-adrenoceptor expression and β-actin. Results (mean ± SEM) are expressed as a ratio of the signal obtained for each protein and the signal obtained for β-actin. *P < 0.05 SO vs. LC rats (Student’s t-test). (**B**) Effect of liver cholestasis on ROCK1, ROCK2, P-MYPT and MYPT expression. The blot is representative of six separate segments from each group. HeLa cell lysates were used as a positive control. Lower panel shows relation between ROCK1, ROCK2, P-MYPT or MYPT expression and β-actin. Results (mean ± SEM) are expressed as a ratio of the signal obtained for each protein and the signal obtained for β-actin. *P < 0.05 SO vs. LC rats (Student’s t-test).

Alpha1-adrenoceptor expression was similar in segments from both groups, while alpha2B-adrenoceptor expression was greater in arteries from LC animals (Fig. 3A).

Both ROCK1 and ROCK2 expressions were similar in arteries from the two rat groups, as well as MYPT expression, while the phosphorylation of MYPT in Thr 696 was higher in arteries from LC rats (Fig. 3B).

**Figure 4:**
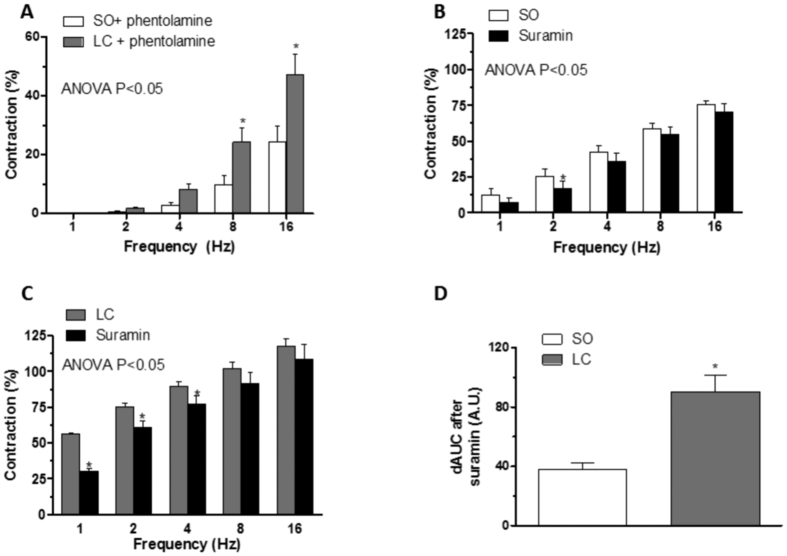
Effect of liver cholestasis on ATP function. (**A**) Remnant EFS-induced vasoconstriction after preincubation with phentolamine. Effect of preincubation with 0.1 mmol/L suramin on vasoconstrictor response induced by EFS in endothelium-denuded mesenteric segments from Sham-operated (SO, **B**) and liver cholestasis (LC, **C**) rats. Results (mean ± S.E.M.) were expressed as a percentage of the initial contraction elicited by KCl. n = 6-8 animals per group. *P < 0.05 (Fisher *post hoc* test) (**D**) Differences of area under the curve (dAUC) in the absence or presence of 0.1 mmol/L suramin. *P < 0.05 SO vs. LC rats (Student’s t-test). dAUC values are expressed as arbitrary units.

When analysing the remnant phentolamine-resistant contractile response, we observed that it was greater in mesenteric segments from LC animals (Fig. 4A). Preincubation with non-specific P2 purinergic receptor antagonist suramin (0.1 mmol/L) decreased EFS-induced vasoconstrictor response in segments from both groups, but more markedly in arteries from LC animals (Fig. 4B–D). The combined preincubation with phentolamine plus suramin practically abolished EFS-induced vasoconstriction in segments from both experimental groups (Table 2). Related to these results, the EFS-induced ATP release was greater in mesenteric segments from LC animals (Table 3).

### Participation of the nitrergic component in vascular responses to EFS

**Figure 5:**
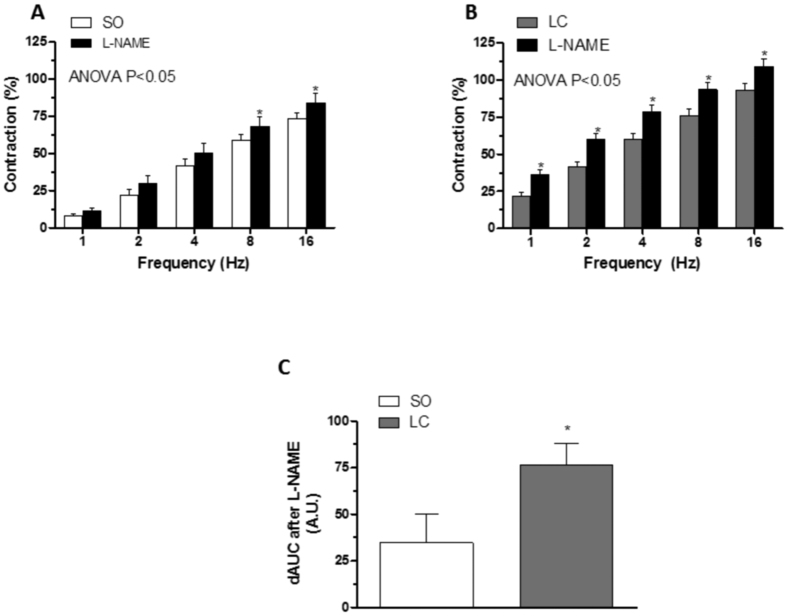
Effect of liver cholestasis on nitrergic innervation function. Effect of preincubation with 0.1 mmol/L L-NAME on the vasoconstrictor response induced by EFS in mesenteric segments from Sham-operated (SO, **A**) and liver cholestasis (LC, **B**) rats. Results (mean ± S.E.M.) are expressed as a percentage of the previous contraction elicited by KCl. n = 6-8 animals per group *P < 0.05 (Fisher *post hoc* test). (**C**) Differences of area under the curve (dAUC) in the absence or presence of 0.1 mmol/L L-NAME; dAUC values are expressed as arbitrary units.*P < 0.05 SO vs. LC rats (Student’s t-test).

**Figure 6:**
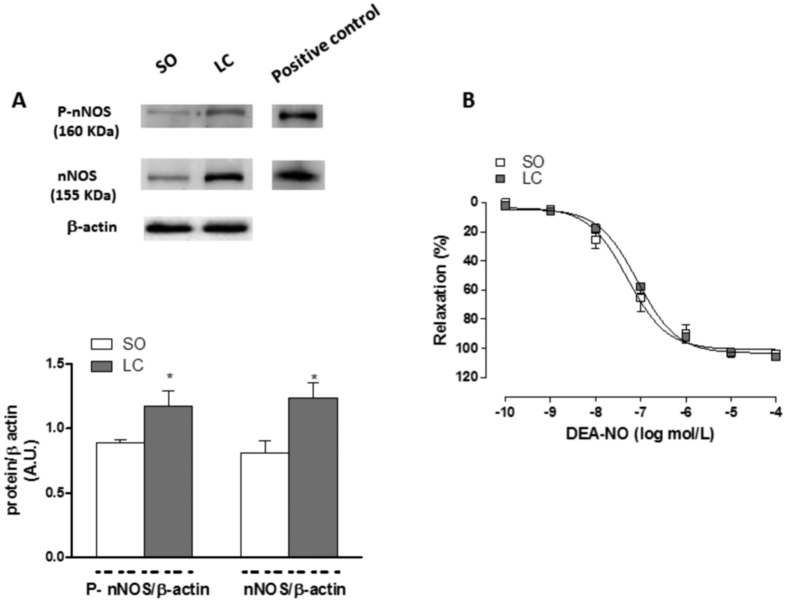
Effect of liver cholestasis on neuronal nitric oxide synthase expression and activation, and on DEA-NO-induced vasodilation. (**A**) Effect of liver cholestasis on nNOS and P-nNOS expression. The blot is representative of six separate segments from each group. Rat brain homogenates were used as a positive control. Lower panel shows relation between nNOS or P-nNOS expression and β-actin. Results (mean ± SEM) are expressed as a ratio of the signal obtained for each protein and the signal obtained for β-actin. *P < 0.05 SO vs. LC rats (Student’s t-test). (**B**) Vasodilator response to NO donor DEA-NO in NA-precontracted mesenteric segments from Sham-operated (SO) and liver cholestasis (LC) rats. Results (mean ± S.E.M.) are expressed as a percentage of the previous tone elicited by exogenous NA. n = 7 animals per group.

Preincubation with unspecific NOS inhibitor L-NAME (0.1 mmol/L) significantly increased the EFS-contractile response in endothelium-denuded segments from both groups (Fig. 5A,B). This increase was greater in segments from LC animals (Fig. 5C). EFS induced nitrite release in segments from both groups. This release was higher in LC mesenteric segments (Table 3). Both the expression and the phosphorylation of nNOS in Ser1417 were increased in mesenteric homogenates from LC animals compared to expression in homogenates from the SO group (Fig. 6A). LC did not alter the vasodilator response to DEA-NO (NA pre-contraction: SO: 1033 ± 74.1 mg; LC: 1046 ± 65.4 mg; P > 0.05) (Fig. 6B).

### Participation of the sensory component in vascular responses to EFS

**Figure 7:**
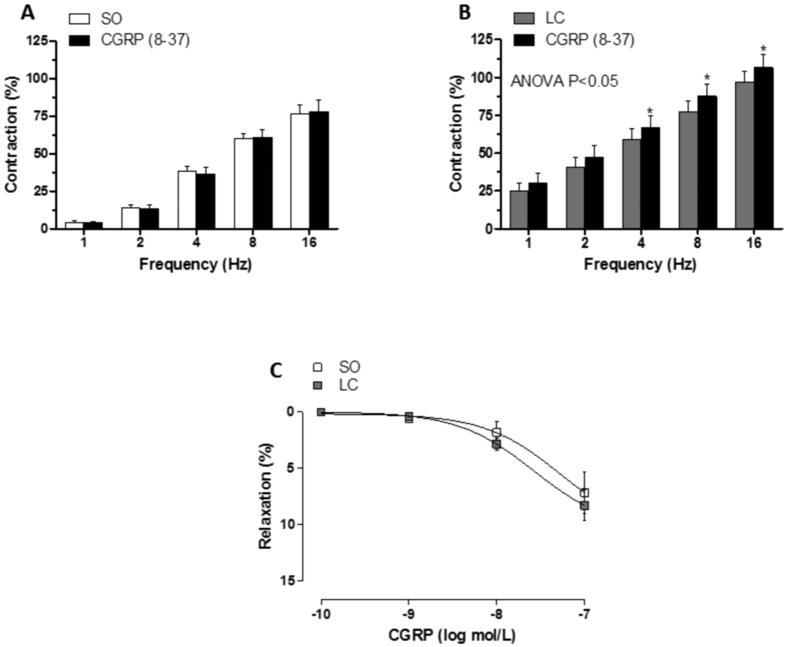
Effect of liver cholestasis on sensory innervation function. Effect of preincubation with 0.5 μmol/L CGRP (8-37) on the vasoconstrictor response induced by EFS in mesenteric segments from Sham-operated (SO, **A**) and liver cholestasis (LC, **B**) rats. Results (mean ± S.E.M.) are expressed as a percentage of the previous contraction elicited by KCl. n = 8 animals per group. *P < 0.05 (Fisher *post hoc* test) (**B**) Vasodilator response to exogenous CGRP in NA-precontracted mesenteric segments from SO and LC rats. Results (mean ± S.E.M.) are expressed as a percentage of the previous tone elicited by exogenous NA. n = 6 animals per group.

Preincubation with the CGRP receptor antagonist CGRP (8–37) (0.5 μmol/L) increased the EFS-induced contraction only in segments from LC animals (Fig. 7A,B). Vasodilator response to CGRP was similar in mesenteric segments from both experimental groups (NA precontraction: SO: 1095 ± 98.7 mg; LC: 1123 ± 62.1 mg; P > 0.05) (Fig. 7C). EFS-induced CGRP release was greater in segments from LC animals (Table 3).

## Discussion

The present study in an experimental model of obstructive LC shows an increase in EFS-induced vasoconstriction in superior mesenteric artery that is the net effect of 1) increased sensitivity to NA through enhanced alpha 2B adrenoceptor expression and ROCK activity, and augmented ATP release, and 2) elevated neuronal NO and CGRP releases from the nitrergic and sensory innervations, respectively.

As we have previously described in this experimental model, LC animals develop liver fibrosis and hepatomegaly, together with a mean arterial pressure decrease, PH, enlarged spleen and collateral portosystemic circulation, as well as hepatomegaly, liver encephalopathy and ascites^38^,^39^,^40^; these alterations make this an appropriate experimental model for studying alterations associated to decompensated liver cirrhosis.

The maintenance and worsening of PH during the progression of cirrhosis is due to the development of two synergistic mechanisms: increased intrahepatic vascular resistance and increased splanchnic blood flow. The characteristic decrease in splanchnic bed vascular resistance observed in liver cirrhosis is associated to modifications in both endothelial and/or neuronal function^41^. Focussing on the latter, the decreases in EFS-induced contraction reported in mesenteric segments from rats subjected to different mild liver cirrhosis models have been attributed to altered sympathetic, nitrergic and sensory function^26^,^27^,^28^,^32^,^42^. However, in this model of decompensated LC we have observed an augmented EFS-induced contractile response in endothelium-intact mesenteric segments. A possible explanation for the observed differences is that LC alters the intrinsic vascular contractile machinery. However we observed that the vasoconstrictor response after exposition to a depolarizing solution of KCl was not different in segments from the two experimental groups, which ruled out this possibility and contrasted to previous reports showing hypo reactivity to KCl in cirrhotic animals^43^.

Several groups have described time-dependent alterations in endothelial function in PH and liver cirrhosis^12^,^13^,^14^,^15^. Endothelium-derived factors are known to affect the response to several vasomotor substances, including neurotransmitters^44^,^45^,^46^,^47^. To determine a possible different influence by endothelium in decompensated cirrhosis, we compared EFS-induced vasoconstriction in endothelium-intact and endothelium-denuded segments. Endothelium removal increased vasoconstrictor response to EFS similarly in both study groups, indicating that the role of endothelium in modulating the EFS vasoconstrictor response is similar in both control and LC animals and suggesting that the differences in EFS-induced vasoconstriction would be due to neuronal alterations. This was confirmed by the fact that the nerve impulse blocker TTX abolished EFS-induced vasoconstriction in segments from both SO and LC animals. Taken together, these data indicate that the increased contractile response observed in LC segments is due to modifications in perivascular innervation function and produced by an endothelium-independent mechanism. We therefore performed the following experiments in endothelium-denuded mesenteric segments.

Several studies have shown that cirrhosis produces alterations in perivascular innervation components. Regarding sympathetic function, both increases and decreases in sympathetic discharge have been described in cirrhosis^30^,^42^,^48^. Thus, our next objective was to analyse the possible participation of sympathetic innervation in the increased vasoconstrictor response observed in LC rats. After incubating segments with the alpha-adrenoceptor antagonist phentolamine, we observed that the vasoconstrictor response elicited by EFS was significantly reduced in segments from both SO and LC animals. This decrease was higher in LC rats, suggesting a different participation by the adrenergic component in the two experimental groups. This effect could be associated to either an alteration in NA release and/or NA-induced vasoconstriction. Although increases and decreases in NA release have both been reported in cirrhosis^27^,^29^,^48^, in our experimental conditions we observed no differences in EFS-induced NA release. Regarding vasoconstrictor response to exogenous NA, previous reports showed both hypo- and hyper-reactivity to NA in mesenteric vessels from cirrhotic animals^12^,^26^,^49^,^50^, the difference depending on the time after cirrhosis onset. In the present study, we found increased NA-induced vasoconstriction in segments from LC animals. This vasoconstrictor response is largely mediated by activation of post-synaptic alpha1 and alpha 2B adrenoceptors in this vascular bed^51^,^52^. We found no alterations in alpha 1 adrenoceptor expression, as previously described^53^, while alpha 2B expression was increased in mesenteric segments from LC animals. Among the components of the signal transduction pathway activated by alpha adrenoceptors, ROCK plays a relevant role in inducing vasoconstriction. Hyperactivation of the ROCK pathway has been reported to enhance phosphorylation and subsequent inactivation of MYPT and this leads to smooth muscle cell contraction^54^. In decompensated LC, we observed no differences in ROCK1 and ROCK2 isoform expression. Nevertheless, ROCK activity was greater in LC animals, since we observed an increase in the phosphorylation of MYPT. This result indicates that ROCK pathway hyperactivity is one of the possible underlying mechanisms responsible for increased NA-induced constriction and, consequently, for the augmented adrenergic function observed in decompensated LC. This result contrasts with observations in mild liver cirrhosis, where the characteristic splanchnic hypocontractility to NA is produced by a downregulated and defective ROCK activation^55^, and reinforces the idea of a rearrangement in the participation of the different vasoactive factors over time.

Sympathetic vasomotor reflexes have been described as involving simultaneous NA and ATP release, both acting on vascular smooth muscle cells^56^,^57^,^58^. The importance of ATP as a functional sympathetic neurotransmitter in blood vessels depends on the species, the vascular bed, the type of blood vessel and the pathology analysed^59^. In this sense, changes in purinergic cardiovascular signalling have been described in liver pathologies^60^,^61^. We have observed a substantial phentolamine-resistant contractile response, greater in mesenteric segments from LC rats, which was abolished after adding the P2-purinoceptor antagonist suramin. What is more, suramin alone also decreased EFS-induced vasoconstriction in both rat groups, but to a greater extent in arteries from LC animals. Both results indicate a greater participation by the sympathetic co-transmitter ATP in LC rats. Purinergic contribution has been reported to depend on previous vascular tone, and is higher when tone is increased^37^,^57^,^59^,^62^,^63^,^64^. Taking this into account, the increased vasoconstrictor response to EFS in LC rats could be responsible for the enhanced ATP release observed in these animals. We have demonstrated that the alterations in NA and ATP releases in this artery not only appear in the same way, as previously described^57^,^58^, but also in the opposite sense, or even one changing and other remaining unaltered^37^,^62^,^64^, indicating a complex interaction between the two neurotransmitters that would depend on the pathophysiological situation. These data show that greater NA-induced vasoconstriction, through augmented alpha 2B adrenoceptor expression and enhanced ROCK activity, and increased ATP release contribute to the increased sympathetic participation and, consequently, to the enhanced EFS-induced vasoconstriction in LC animals.

The pivotal role played by endothelial NO in the high splanchnic vasodilation observed in PH and liver cirrhosis has been widely demonstrated^7^,^65^, and it either increases vasodilation by itself, or it leads to a hypo responsiveness to constrictor factors such as NA^14^. Regarding neuronal NO, earlier reports have shown increased levels in mesenteric arteries and veins in both PH and compensated cirrhosis, and that this participation decreases through time^21^,^66^. To evaluate possible alterations in nitrergic innervation in LC, segments from both experimental groups were incubated with non-specific NOS inhibitor L-NAME. We observed increased EFS-induced constrictor response in segments from both groups, but the response was stronger in arteries from LC animals, indicating a greater role by nitrergic innervation. This increase in nitrergic participation can be due to an alteration in the synthesis of and/or the vasodilator response to nitrergic neurotransmitter NO. The analysis of EFS-induced nitrites, the stable end metabolic product of NO, showed an increase in segments from LC animals, which agrees with the augmented nNOS expression and phosphorylation that we observed. The vasodilator response to the NO donor DEA-NO was similar in both SO and LC rats, contrasting with the increase observed in other cirrhosis models^67^,^68^. Together, these observations indicate that the nitrergic innervation participation in segments from LC rats is enhanced through increased NO release. The great amount of interactions described between NA, ATP and NO highlight the complexity of the relationship between these vasoactive factors. Thus, the simultaneous increase in ATP and NO releases observed in this artery in this and other studies^37^,^65^ agrees with previous observations in endothelium and central nervous system^69^,^70^,^71^. However, a direct relationship between both neurotransmitters does not always appear, since we have also observed increased ATP together with decreased NO release^62^. Additionally, we have previously reported that modifications in NO release are independent of alterations in NA vasoconstriction^37^,^63^,^64^,^72^. Once again, these results seem to confirm that the possible interaction between the different components of perivascular innervation depends on the pathophysiological situation under study, and highlight the need for studying innervation function in different PH and cirrhosis models.

A recent review reports that, despite the wide distribution of sensory innervation in different vascular beds, sensory participation is only revealed in adverse cardiovascular events, and acts as a compensating and/or protector mechanism^73^, in agreement with previous reports from our groups^21^,^32^,^72^. Here we observed that preincubation with the CGRP antagonist CGRP (8-37) did not modify the vasoconstrictor response to EFS in mesenteric rings from SO rats, although it increased it in segments from LC rats. These results indicate a role for sensory innervation in EFS-induced contraction only in decompensated cirrhosis, as previously observed in the compensated phase of this pathology^32^. Our next objective was to determine whether this participation was associated with an increase in CGRP release and/or vasodilation. EFS-induced CGRP release was higher in mesenteric segments from LC rats. Additionally, exogenous CGRP produced no differences in the vasodilation in segments from either rat group, ruling out alterations in sensitivity to CGRP. This result contrasts with previous observations in compensated liver cirrhosis, where an increased CGRP vasodilator response was observed in this artery^32^, once more reinforcing the idea that the mechanisms implicated in the alterations of splanchnic hyperdynamic circulation depend on the time since onset and on the severity of the pathology, and that CGRP only has a relevant role in pathological situations.

Among other causes, vascular complications associated to PH and liver cirrhosis are due to inflammatory mechanisms^38^, for instance, cyclooxygenase (COX) is overexpressed in smooth muscle cells from different vascular beds^74^,^75^. We have previously described that the COX derivates TXA_2_ and PGI_2_ play a role in modulating sympathetic and nitrergic function in this artery. In this sense, an increase in COX activity and subsequently TXA_2_ expression leads to augmented NA vasoconstriction^76^. Additionally, over-release of endogenous PGI_2_ enhances neuronal NO release^77^. Thus, it is possible that COX derivatives could modulate the innervation function in decompensated LC.

Altogether, the results of the present study demonstrate that, in superior mesenteric arteries, decompensated LC produces a) increased vasoconstrictor response to NA, due to enhanced alpha 2B adrenoceptor expression and ROCK activity; b) augmented ATP release; c) raised release of the nitrergic neurotransmitter NO; and d) enhanced release of the sensory neurotransmitter CGRP. The net effect of these alterations is an increase in the EFS-induced vasoconstrictor response and this might be a counteracting mechanism to the increased splanchnic vasodilation observed in the last phases of decompensated liver cirrhosis.

## Additional Information

**How to cite this article**: Sastre, E. *et al*. Decompensated liver cirrhosis and neural regulation of mesenteric vascular tone in rats: role of sympathetic, nitrergic and sensory innervations. *Sci. Rep.* **6**, 31076; doi: 10.1038/srep31076 (2016).

## Figures and Tables

**Table 1:** Effect of liver cholestasis on body weight (BW), body weight gain (BWG), portal pressure (PP), liver weight to body weight ratio (LW/BW), spleen weight to body weight ratio (SW/BW) and ascetic fluid extravasation in Wistar rats.

	BW (g)	BWG (g)	PP (mm Hg)	LW/BW (%)	SW/BW (%)	Ascetic fluid (mL)
SO	412.5 ± 9.3	43.8 ± 5.6	8.3 ± 2.1	3.06 ± 0.06	0.25 ± 0.03	—
LC	305.4 ± 10.5*	22.4 ± 6.1*	14.7 ± 2.4*	5.74 ± 0.34*	0.80 ± 0.19*	6.4 ± 1.4

Results are expressed as means ± S.E.M. ^∗^P < 0.05 versus SO. n = 10 animals each group.

**Table 2:** EFS-induced contraction after preincubation with 0.1 μmol/L TTX or 1 μmol/L phentolamine plus 0.1 mmol/L suramin, in mesenteric segments from SO and LC rats.

	1 Hz	2 Hz	4 Hz	8 Hz	16 Hz
SO	10.1 ± 1.4	21.7 ± 2.3	39.7 ± 2.8	59.2 ± 2.8	77.1 ± 2.9
+ TTX	0	0	0	0.5 ± 0.17*	1.3 ± 0.31*
+phentolamine + suramin	0	0	0.2 ± 0.12*	0.8 ± 0.20*	2.5 ± 0.23*
LC	20.7± 3.1	35.9 ± 4.2	52.9 ± 4.7	68.8 ± 4.8	86.4 ± 4.8
+ TTX	0	0	0	0.17 ± 0.09*	1.57 ± 0.14*
+phentolamine + suramin	0	0	0.1 ± 0.03*	1.2 ± 0.5*	2.6 ± 1.5*

Results (means ± SEM) are expressed as percentages of the response elicited by 75 mM KCl. Zeros are used when contraction was not detected. *P < 0.05 vs. conditions without drug. n = 6-10 animals each group.

**Table 3:** Effect of liver cholestasis (LC) on EFS-induced NA, ATP, nitrite and CGRP release.

	NA release (pg/mL mg tissue)	ATP release (pmol/mL mg tissue)	Nitrite release (pmol/mg tissue)	CGRP release (pg /mL mg tissue)
SO	0.87 ± 0.09	11.8 ± 3.1	1.3 ± 0.4	0.03 ± 0.01
LC	0.81 ± 0.31	68.1 ± 14.5*	15.4 ± 4.6*	0.21 ± 0.02*

Results were calculated by subtracting basal release from that evoked by EFS, and expressed as means ± S.E.M. ^∗^P < 0.05 versus SO. n = 10 animals each group.
